# Retraction: Persistent childhood primitive reflex reduction effects on cognitive, sensorimotor, and academic performance in ADHD

**DOI:** 10.3389/fpubh.2025.1633428

**Published:** 2025-06-03

**Authors:** Frontiers Editorial Office

The journal retracts the 17 November 2020 article cited above.

Following publication, concerns were raised regarding the scientific validity of the article. An investigation was conducted in accordance with Frontiers' policies. The authors failed to provide a satisfactory explanation and as a result, the conclusions of the article have been deemed unreliable and the article has been retracted. This retraction was approved by the Chief Editors of Frontiers in Public Health and the Chief Executive Editor of Frontiers. The authors do not agree to this retraction.

